# How is diagnostic uncertainty communicated and managed in real world primary care settings?

**DOI:** 10.1186/s12875-024-02526-x

**Published:** 2024-08-12

**Authors:** Jessica Russell, Laura Boswell, Athena Ip, Jenny Harris, Hardeep Singh, Ashley N. D. Meyer, Traber D. Giardina, Afsana Bhuiya, Katriina L. Whitaker, Georgia B. Black

**Affiliations:** 1 Wolfson Institute of Population Health, Queen Mary University of London, London, UK; 2 School of Health Sciences, The University of Surrey, Surrey, UK; 3 Center for Innovations in Quality, Effectiveness and Safety (IQuESt), Michael E. DeBakey VA Medical Center, Department of Medicine, Baylor College of Medicine, Houston, TX USA; 4 General Practitioner, Cancer GP lead for North Central London Cancer Alliance, London, UK

**Keywords:** Health communication, Primary care, Video recording, Patient safety, Consultation, Safety netting, Uncertainty

## Abstract

**Background:**

Managing diagnostic uncertainty is a major challenge in primary care due to factors such as the absence of definitive tests, variable symptom presentations and disease evolution. Maintaining patient trust during a period of investigative uncertainty, whilst minimising scope for diagnostic error is a challenge. Mismanagement can lead to diagnostic errors, treatment delays, and suboptimal patient outcomes.

**Objective:**

Our aim was to explore how UK primary care physicians (GPs) address and communicate diagnostic uncertainty in practice.

**Design:**

This qualitative study used video and audio-recordings. Verbatim transcripts were coded with a modified, validated tool to capture GPs’ actions and communication in primary care consultations that included diagnostic uncertainty. The tool includes items relating to advice regarding new symptoms or symptom deterioration (sometimes called ‘safety netting’). Video data was analysed to identify GP and patient body postures during and after the delivery of the management plan.

**Participants:**

All patient participants had a consultation with a GP, were over the age of 50 and had (1) at least one new presenting problem or (2) one persistent problem that was undiagnosed.

**Approach:**

Data collection occurred in GP-patient consultations during 2017–2018 across 7 practices in UK during 2017–2018.

**Key results:**

GPs used various management strategies to address diagnostic uncertainty, including (1) symptom monitoring without treatment, (2) prescribed treatment with symptom monitoring, and (3) addressing risks that could arise from administrative tasks. GPs did not make management plans for potential treatment side effects. Specificity of uncertainty management plans varied among GPs, with only some offering detailed actions and timescales. The transfer of responsibility for the management plan to patients was usually delivered rather than negotiated, with most patients confirming acceptance before concluding the discussion.

**Conclusions:**

We offer guidance to healthcare professionals, improving awareness of using and communicating management plans for diagnostic uncertainty.

**Supplementary Information:**

The online version contains supplementary material available at 10.1186/s12875-024-02526-x.

## Background

The management of diagnostic uncertainty is a fundamental challenge in primary care [1]. Uncertainty can arise from a multitude of factors, including a lack of definitive diagnostic tests, variable presentation of symptoms, and limited access to specialist services [2]. Furthermore, the communication of diagnostic uncertainty represents a complex challenge for physicians who must balance patient expectations and potential harm from missing a diagnosis with managing over-referral to specialist services. Diagnostic uncertainty has implications for patient safety as its mismanagement can contribute to diagnostic errors, treatment delays or inadequacies, and suboptimal patient outcomes [1, 2]. 

A critical aspect is effective communication of areas of potential uncertainty, both within the healthcare team and with patients. Diagnostic uncertainty is not always discussed with patients in practice, with potential ethical implications for patient autonomy and safety [3, 4]. Even when discussed, the linguistic expressions of uncertainty can vary [5], and the reactions and experiences of patients may differ based on cultural sensitivities and individual preferences [6]. 

Reviews of empirical literature have considered how best to manage diagnostic uncertainty, recommending strategies such as using patient-centred communication, acknowledging uncertainty, creating diagnostic ‘safety nets’, and using diagnostic reasoning strategies [6, 7]. Even when such strategies are used, they may not be done well. While a variety of methods are used to appraise management of diagnostic uncertainty, (including surveys and qualitative interviews with clinicians; video or audio recorded consultations and experimental techniques [2, 8, 9]) these can struggle to capture the dynamic nature of diagnostic uncertainty [9]. As a result, there is little empirical evidence on how uncertainty is or should be communicated in primary care.

The practice of creating ‘safety nets’ (commonly referred to as “safety netting” is the UK) is a potentially beneficial method for managing uncertainty [10]. A safety net is a management plan discussed with the patient to provide specific guidance for uncertain situations, including what to expect and possible outcomes e.g. when and how to seek further medical attention if their symptoms persist or worsen or new symptoms arise [11–13]. It can mitigate the risk of delayed treatment (and subsequent potentially sub-optimal outcomes)by equipping patients with information on scenarios that require swift action. In addition to advice, it can take the form of safety netting systems e.g. automated reminders to attend specialist appointments [2]. Effective safety netting acknowledges patients’ information needs, acceptance and understanding [14], but is challenged by multiple presenting problems, general practitioner (GP) workload and remote consultations [13–15].

Understanding how diagnostic uncertainty is managed in primary care is essential for improving the safety and efficiency of diagnosis [9]. Using video data of consultations in primary care, this paper presents an analysis of the management strategies used by primary care physicians in the UK to address uncertainty and their impact on patient care.

## Methods

### Methodological approach

This is a secondary qualitative analysis study applying a validated structured tool [16] based on a dataset of video and audio-recorded GP-patient consultations collected in Surrey and London, UK in 2017–2018. The tool was designed to record the characteristics of safety netting delivery using video data. Our recordings were collected from 7 GP practices, with 10 participating GPs. The primary aim of data collection was to understand doctor-patient communication around the significance of persistent or new presenting problems and its potential impact on timely cancer diagnosis. Full information on data collection, participant recruitment and ethical approval for this dataset can be found in Amelung et al. [17]. 

### Sample

The total dataset consisted of 200 complete videos, from which we derived a sample of 90 eligible consultation videos for this study according to the following inclusion criteria: all patient participants had a consultation with a GP and had (1) at least one new presenting problem or (2) one persistent problem that was undiagnosed. We selected patients aged 50 or older for our study, as this age group has a higher incidence of cancer than younger populations (see Table 1) [18]. All patient participants and GP participants gave informed consent to have their consultation video-recorded.

**Table 1:** Patient and doctor characteristics

	Patients	General practitioners
Age (*average; min-max*)	67 (50 − 9)	48.2 (32–60)
Gender		
Male	37	7
Female	53	3
Ethnic origin *n* (%)		
White/White British/White Other	69	9
Asian/Asian British	3	1
Black/African/Caribbean/Black British	8	0
Other ethnic group	4	0
No information re ethnicity	6	0
Highest education qualification		
Degree or higher degree	20	
Below degree level	56	
No formal qualification	14	
Years since medical accreditation; mean (min-max)		15.9 (2–32)
Accredited doctor trainer		6

### Data collection

Video footage of GP-patient consultations was collected using digital and audio-video recording devices. Consultations were recorded in four different GP practices across London and the South East of the United Kingdom.

### Ethical considerations

Patients were approached in the practice waiting room and given information about the study to read. Patients and GPs both provided written informed consent to participate in the study and the video recording. All data were stored in a secure manner, and access was restricted to the research team to ensure confidentiality. Audio files were transcribed verbatim and all personally identifiable information was anonymized to protect privacy. Ethical Approval was obtained from London Chelsea Research Ethics Committee (17/LO/0270).

### Data Analysis

#### Coding of verbatim transcripts

A tool for coding safety netting behaviours in primary care [16] was applied to the verbatim transcripts by JR and discussed regularly with GB. The tool contains codes relating to:

- Problem administration - how many presenting problems, nature of presenting problem.
- Diagnostic context - whether the GP communicates a diagnosis.
- Follow up codes - whether another consultation is planned.
- Nature of safety netting advice.
- Initiation of safety netting advice.
- Delivery of safety netting.
- Conditions/symptoms in safety netting advice.
- Actions advised in safety netting advice.
- Patient response to safety netting.
- Communication format - writing, verbal.
- Safety netting documentation.

A full table of the coding tool and response options is freely available from the original Edwards et al. publication [16]. The verbatim text data were imported and managed in Excel. We modified the tool to capture management of diagnostic uncertainty more widely. The process for this began with identifying instances of diagnostic uncertainty for discussion between JR and GB. After preliminary rounds of coding applying the tool, we added the following additional codes to enable more in depth exploration of *how* diagnostic uncertainty was managed in primary care consultations (see Supplementary file 1).

#### Coding of video data

After the modified safety netting tool had been applied to the verbatim transcripts, the video data was analysed. Each video was imported into NVIVO qualitative software and the safety netting episode was analysed; deductive codes were generated to identify behavioural aspects of how the GP delivered the management plan for uncertainty (e.g. was the GP facing the patient/computer), how the patient responded behaviourally (e.g. no behavioural response, nodding, shaking head) and what the GP and the patient did immediately after delivering the management plan (see Supplementary file 2). These codes were applied to the video data, generating inductive codes during the coding process. The two researchers (JR and LB) coded all videos, compared results and discussed any differences in coding.

Overall, the combination of participant selection, data collection, and analysis methods were designed to enable the study to capture authentic GP and patient interactions in a naturalistic primary care setting. The use of video data provided a unique opportunity to explore the complexity of verbal and non-verbal communication during primary care consultations.

## Findings

### Areas that provoke diagnostic uncertainty for GPs

#### Undifferentiated and/or persistent symptoms

GPs frequently advised patients to monitor persistent or worsening symptoms (*n* = 32, 50%), including current symptoms, new symptoms and symptoms that may worsen over time (*n* = 13, 20%). This ‘test of time’ approach included the GP’s suspected diagnosis and a plan for how the persistence of the problem would affect their diagnostic management.

For example, in response to a patient presenting with redness on their shin bone following a fall, a GP explained that the redness could be inflammation or infection and asked the patient to monitor the redness on their leg:

*“But what I would encourage you to do is.is to have a mental note…and can I just sort of point out what I’m doing…is that*,* is this redness here .I think if that begins to spread further I think we…we’d call this cellulitis… there’s some good bone swelling and inflammation and you’re right*,* you probably bashed the bone…” [GP83]*.

#### Prescription of medication

GPs mainly expressed advice about monitoring symptoms when no treatment was prescribed for the presenting problem and there were no further investigations (*n* = 29, 60%). When GPs expressed uncertainty alongside prescribed home treatment, it was mainly related to whether patients were on the correct treatment or whether the treatment they prescribed was effective (*n* = 18, 28%).

*GP: Could you try the cream for a maximum of 2 weeks and…*.

*Patient: Okay.*

GP: *…if it’s not getting better you come back and see us.* [GP291]

*“If these drops don’t seem to work there are other brands*,* other types of drops with slightly different ingredients*,* so if after one to two weeks it’s not feeling better just tell us and we’ll swap to a different antibiotic brand*,* okay?” [GP61]*.

In our dataset, there were no instances where GPs expressed uncertainty about potential side effects of the treatment they prescribed (e.g. if [specified symptoms] occur after using [medication] take [action]). In only two cases, the GP expressed a plan to cover uncertainty regarding whether the patient would adhere to their advice, and offered encouragement to the patient to either take prescribed medication or to attend an investigative procedure.

*GP: So even if when you’re taking the tablets all the symptoms get better and you think oh it’s all better now*,* I don’t need to really go for that horrible camera test…*.

*Patient: Yeah.*

*GP: …you really should because we want to be completely sure about what’s going on inside the oesophagus*,* why the gullet isn’t squeezing down*,* things down properly and what’s causing all this extra acid in the first place*,* okay? [GP158]*

#### Potential administrative errors in the diagnostic process

GPs also expressed uncertainty when they had administrative concerns about patients accessing investigations or secondary care appointments following their primary care appointment. They were concerned that the results of hospital investigations would get lost and patient appointment letters would not arrive.

In this example the patient’s investigative results from secondary care had never reached their GP. The GP referred the patient for a repeat test and asked the patient to request a copy of their own results as a safety net to this error happening again:

*“Well the only thing I say this time is…request that you ask for a copy to be given directly … for you to give directly to me (points towards themself) because they will give you a copy of it and then we will be sure if it’s in your hands.” [GP143]*.

Similarly, another GP advised the patient to contact the GP surgery if their Urgent Suspected Cancer referral appointment letter did not arrive.

### Verbal communications by GPs to patients to manage uncertainty

Plans to manage uncertainty were initiated by GPs with only 8 cases of patients initiating these discussions. It was almost exclusively the responsibility of the patient to take action for any required further consultations (n = 58, 91%).

*“Well*,* if you’re not back to completely normal in another week will you let me know?” [GP105]*.

GPs predominantly delivered advice about managing uncertainty during the treatment planning phase of the consultation (n = 50,83%) or at the closing of the consultation (n = 13, 20%). This was invariably delivered as a plan, with a request only for confirmation from the patient:

*“I mean*,* if your hair really starts falling out… it will probably be worth doing those blood tests again*,* that’s the only sort of thing*,* that’s … worth doing here really*,* yes?” [GP228]*.

GPs mostly followed the communication format of providing a list of symptoms for patients to monitor or be aware of, followed by an accompanying action for patients to take (n = 57, 89%). In a minority of cases, GPs did not advise any accompanying action for what the patient should do if the symptom arose or persisted (n = 9, 14%). When GPs did advise, the action to take was invariably to return to them personally or to their GP surgery (n = 45, 64%). In 10 cases they directed patients to other services, such as emergency services.

*“And I think if this cough has not gone back to how it normally is in another week and you’re not feeling fully better I’d like you to come and see me*,* and I think we should think about sending you up for some more tests.” [GP105]*.

There was substantial variation as to whether GPs also provided patients with a timescale for taking the action advised, with no timescale provided in just over half of cases (n = 36,56%).

*“And we’ll leave the antibiotics for time being… unless*,* you know*,* it gets worse again.” [GP269]*.

GPs tended to use neutral (*n* = 25,39%) or weak language (*n* = 30, 47%) to endorse their advice. Neutral language includes wording such as “come back and see me” or “pop back”, where weak endorsement is expressed as “you *could/can* come back”.

### Communication following the management plan for uncertainty

GPs predominantly communicated the management plan facing the patient, although in some cases they either faced the computer or turned their body back and forth between the computer and the patient. In nearly all cases, as soon as the management plan was delivered, the GP’s body position changed to either turning to face the computer or preparing to usher the patient out of the consultation. This was also reflected verbally; at the end of delivering the management plan, GPs either stopped talking in order to work on their computer, discussed something else (e.g. plans for patient’s investigative tests) or listened to the patient. Patients sat in silence whilst the management plan was delivered, displaying behaviours that signalled understanding or agreement (such as nodding or *“uh huh”* phrases) or neutral behaviours.

No patient actively disagreed with the plan, one patient used quiet sarcasm, muttering *“fab”*. At the end of the delivery of the verbal plan patients either changed the topic, sat silently whilst the GP worked on the computer or started to collect their possessions in order to leave the consultation.

## Discussion

The results of this study provide insights into when and how GPs communicate plans to manage diagnostic uncertainty during primary care consultations. A range of management plans were identified, including those related to diagnostic and treatment uncertainties: (1) patients who were not prescribed treatment and needed to monitor existing symptoms or potential new symptoms; 2) patients who were prescribed treatment and needed to monitor symptoms to assess treatment efficacy and 3) risks to the diagnostic process due to administrative problems in the system. There were no management plans for potential side effects of treatment and only one case of using a management plan to mitigate potential lack of adherence. Our findings suggest that the specificity of uncertainty management plans varied, with some GPs providing more detailed information than others regarding required actions, including timescales for when these actions should be taken. We identified that the transfer of responsibility was often delivered rather than negotiated, with patients invited solely to confirm acceptance of the plan before the discussion ended. After the management plan had been delivered, both GPs and patients behaviourally adopted postures that indicated that either discussion or the consultation had ended. We have made some recommendations for the communication of diagnostic uncertainty plans based on our findings (see Table 2).

**Table 2:** Recommendations for practice

Recommendations	Example
Be specific about time frames to ask patients to return.	*“The cough should be better in 2 weeks from now*,* if not please rebook with a GP to follow this up.”*
When a test/investigation is advised, take steps to ensure its completion, including encouraging adherence and organising follow up or rebooking if required.	*Two potential actions for GPs to take post consultation in this scenario: Mrs X has cognitive impairment and if she has not returned her qFIT test in 2 weeks*,* either* 1) *consider booking a follow up appointment in advance* 2) *or adding her to a safety netting system that will enable a recall for her if the test is not done.*
Patients should be informed about what to do if medication does not work and outline potential side effects.	*“The nature of your symptoms suggest a bacterial infection and I would advise taking a course of antibiotics to clear this infection. You would take one capsule three times a day and expect that within 24–48 h your fever should have settled. Like most drugs*,* there are side effects. Common ones include diarrhoea*,* vomiting and skin reactions. If you are unable to cope with the side effects then do come back to us.”*
A management plan should be discussed using patient-centered communication, checking that patients are comfortable with any responsibilities after the consultation.	*“At this stage*,* I would like to discuss some options. If I give you my thoughts around the options and then you can come back to me with any questions?* *As mentioned*,* I think constipation is the most likely cause here. I would advise having more fibre in your diet*,* drinking more water and adding a laxative.* *Are you happy with this so far?* *If after these changes you are not improved in the next 3–4 weeks*,* I would like you to book another GP appointment. How do you feel about this plan?”*
Summarise and share your agreed upon management plan and ensure patients have the ability to relay what they understood.	*“We have discussed a range of possible causes for these symptoms at this stage. Our plan is to trial this treatment whilst we wait for the blood tests*,* stool test and ultrasound report to be carried out and reported. I am aware there are a number of actions following this consultation*,* so let’s recap together what has been agreed so we can ensure things go smoothly? I am really happy to share the written plan with you too.”*
Improve administrative processes to reduce the burden on patients and GPs needing to chase results/appointments.	*There are a range of processes that can be used and implementation depends on GP practices’ existing operational structures and staffing and these should be reviewed*: • *Allocate sufficient administrative staff time to ensure completion of investigative procedures*,* including return of test results to GPs* • *Standard operating procedures to help guide staff process admin queries* • *Utilise readily available (funded/free) electronic tools to support you to track at risk patients e.g. E-safety netting systems and templates* • *Promote and support patients to use NHS patient apps to see their records and engage with their care* • *Ensure patients’ contact details are correct e.g. offer online updating links*

### Comparison to previous research

National strategic drivers in the UK such as the NHS Long Term Plan have highlighted patient-led initiatives and patient-activated follow-ups to manage demand for health services, with patients given access to digital records.

We saw that uncertainty caused by administrative gaps that pose risk to diagnostic processes result in physicians transferring responsibility to patients to ensure process completion. This resonates with the growing movement to place patients at the centre of patient safety, with the assumption that patients will check on system performance and quality standards if they know what to expect [19]. We do not know what the impact of being led to expect suboptimal performance in this manner is on patients, and may detrimentally impact patient outcomes due to decreased trust and confidence in their provider. Research has shown that patients who may have limited or fluctuating capacity to self-manage or self-advocate are at greater risk of negative outcomes, particularly people with serious mental health conditions, learning disabilities and autistic people [20–22]. Reasonable adjustments in healthcare settings need to include comprehensive and reliable administrative systems to prevent these patients from losing access to healthcare [23]. 

The NHS Long Term Plan also centres on shared decision-making as part of the consultation model. Consistent with other studies, our findings demonstrate how diagnostic uncertainty is triggered by ambiguity about the underlying cause of patients’ presenting symptoms [3, 24]. Many studies have raised the importance of balancing uncertainty communication with patient-centred strategies. For example, in a realist review of safety netting, recommendations included discussing rather than simply delivering the safety netting plan, with roles negotiated [14]. However, our study demonstrated that in practice, diagnostic uncertainty plans are mainly delivered by the physician, rather than discussed, and frequently terminate the conversation or consultation.

Despite medication safety being repeatedly identified as the predominant patient safety issue in primary care [25–27], relatively few studies have examined the relationship between diagnostic uncertainty and medication prescribing. A retrospective observational study found that diagnostic uncertainty was expressed in 16% of visits, particularly in cases where antibiotics were prescribed [28]. We found no instances of physicians communicating their uncertainty with respect to safety in prescribing medication. This is a missed opportunity, as evidence suggests that patients who are informed about potential side effects of medicine are more likely to take them as recommended [29]. In their Realist Review, Friedemann Smith [14] et al. mapped safety netting to the three most common taught consultation models in medical training, showing that making management plans for diagnostic uncertainty can be integrated into practice rather than being seen as an additional task.

To date, the majority of research has focussed on the content of management plans and their delivery rather than their social function within the primary care consultation. One qualitative study supports our findings that these management plans indicate to patients that the GP is ending a discussion or the consultation. Previous studies indicate that patients retain approximately 40% of information given in a consultation, with factors such as anxiety and age of patient and perceived importance of information resulting in lower recall [30]. If patients deem that they are being dismissed, this may affect their ability to take in information, especially for those who are highly concerned about their health [15, 31]. Further research is also needed to explore how these plans play a role in the doctor-patient relationships e.g. by preserving care continuity (I can return) [32] and potentially relieving immediate anxiety (no/low immediate risk).

Further research is also required to understand patients’ interpretation of safety netting conversations; this includes both comprehension, information retention and the consequences of inferred dismissal or closure. Our adaptation of the Edwards et al. safety netting tool to include other expressions of diagnostic uncertainty may be used in other studies of observational or video data [16]. 

### Strengths and limitations

This study used both inductive qualitative analysis and a validated observational tool to rigorously analyse diagnostic uncertainty management in routine consultations with primary care patients in the UK. The coding of consultations was done with rigour including double coding and the use of a tool with high interrater reliability. The tool was developed specifically for safety netting, which may limit its applicability to diagnostic uncertainty; however, we added unvalidated items in line with our research objective to mitigate this.

Our study is limited by its small sample of primary care consultations in the UK, and by the historical context of using consultations from 2017 to 2018. Primary care practitioners may approach diagnostic uncertainty differently now, particularly since the advent of more widespread electronic safety netting tools and telephone consultations; [33, 34] further research is required to evaluate our proposed recommendations, to assess the uptake of these tools and examine the communication of management plans in a telemedicine context.

We did not measure clinician or patient satisfaction, nor any long term clinical or behavioural outcomes, and therefore cannot comment on the effectiveness of these management plans. The video recording may have influenced physician behaviour; however a methodological review of recording consultations concluded that it does not significantly alter patient or physician behaviour [35]. We did not collect the sample with the aim of analysing diagnostic uncertainty management, therefore this makes it more likely that physicians were acting in a natural manner with no desirability bias.

A limitation of the study is the broad definition of diagnostic uncertainty [2]. To prevent bias incurred from coding in a particular way according to our definitions, we worked with a multi-disciplinary team (including primary care clinicians) in the design, analysis and development of recommendations.

## Conclusion

Diagnostic uncertainty is a fundamental part of primary care practice, requiring careful management to mitigate potential harm from diagnostic errors, treatment delays and suboptimal patient outcomes. Our study has highlighted potential missed opportunities for using management plans, ways to improve management plans and some of the potential social functions these plans can have in the consultation. Our findings offer guidance to healthcare professionals in primary care and other settings, enhancing awareness of how diagnostic uncertainty is conveyed and managed. Our research provides a methodological springboard for future research aiming to clarify the relationship between expressions of diagnostic uncertainty in practice, management strategies and patient outcomes.

### Electronic supplementary material

Below is the link to the electronic supplementary material.

Supplementary Material 1

Supplementary Material 2

## Data Availability

Data are available on reasonable request. Contact Dr Georgia Black on g.black@qmul.ac.uk.

## Declarations

### Patient and public involvement statement

This is a secondary analysis of an existing dataset. For full information on PPI in data collection and analysis see Amelung D, Whitaker KL, Lennard D, et al. Influence of doctor-patient conversations on behaviours of patients presenting to primary care with new or persistent symptoms: a video observation study. *BMJ Qual Saf*. 2020;29(3):198–208.
