# Supplementary material for: How is diagnostic uncertainty communicated and managed in real world primary care settings?
Source: BMC Prim Care. 2024 Aug 12;25:296. doi: 10.1186/s12875-024-02526-x (PMC11318185; doi:10.1186/s12875-024-02526-x)
Supplement: Supplementary file 1 — Supplementary Material 1 [file 12875_2024_2526_MOESM1_ESM.docx]

Supplementary file 1

*Additional codes in relation to how diagnostic uncertainty was managed in primary care*

| **Additional code** | **Description of code** | **Example of verbatim text** | **Example of inductive code** |
| --- | --- | --- | --- |
| Reason for uncertainty management plan | Presenting problem symptoms that are specifically covered by the management plan. Can also include management plans to cover administrative failures. | Patient: Mhm. But nothing more sinister behind it?  GP: I don't think so.. But if- if it persists, or gets worse pop back. | Patient suspected cancer symptom |
| Function of uncertainty management plan | Outcome of management plan if plan is successful | GP: Could you try the cream for a maximum of 2 weeks and...  Patient: Okay.  GP: ...if it’s not getting better you come back and see us. | Ascertain treatment is correct |
| Context of uncertainty management plan | The period that the safety netting covers,  e.g.   - Post consultation, no treatment - Post consultation,GP prescribed treatment - Post consultation, waiting for investigative appointment, - Post consultation, waiting for secondary care appointment | GP: So if you didn't hear anything within 2 weeks, let us know. | Post consultation, waiting for urgent suspected cancer referral |
| Type of uncertainty | The type of uncertainty the GP is creating a management plan to cover   - diagnostic uncertainty - treatment uncertainty - administrative uncertainty | GP: obviously if things are worsening, any change, err, further changes, let me know. | Symptom deterioration |
