# Supplementary material for: How is diagnostic uncertainty communicated and managed in real world primary care settings?
Source: BMC Prim Care. 2024 Aug 12;25:296. doi: 10.1186/s12875-024-02526-x (PMC11318185; doi:10.1186/s12875-024-02526-x)
Supplement: Supplementary file 2 — Supplementary Material 2 [file 12875_2024_2526_MOESM2_ESM.docx]

*Supplementary file 2. Video data codes to capture behavioural responses with respect to diagnostic management plan*

| **Stage of consultation** | **Response options** |  |
| --- | --- | --- |
| GP delivery of management plan | - GP faces computer screen - GP faces patient - GP faces patient: lists advice on fingers - GP intermittently faces computer and patient - GP looks at paper letter - GP off camera, unable to see - GP standing up and faces patient - No video data - Patient initiated management plan |  |
|  |  |  |
|  |  |  |
|  |  |  |
|  |  |  |
|  |  |  |
|  |  |  |
|  |  |  |
|  |  |  |
| Patient’s first response to management plan | - Shows signs of agreeing or understanding (e.g. nodding) - Neutral (no signs of agreeing or disagreeing) - Shows signs of disagreeing (e.g. shakes head, frowns) - Patient interrupts GP - No video data - Patient response is off camera - Patient initiated management plan |  |
|  |  |  |
|  |  |  |
|  |  |  |
|  |  |  |
|  |  |  |
|  |  |  |
| GP posture post management plan | - Remains facing computer - Remains facing patient - Remains facing patient: starts to fill in form - Remains facing patient: starts preparing swabs - Remains standing - Starts to turn away but stops - Turns to face computer - Turns to face computer, then gets up to face the door - Turns to face patient - Turns to face door - Turns to show patient content of paper - Turns to tidy desk   Off camera   - No video data |  |
|  |  |  |
|  |  |  |
|  |  |  |
|  |  |  |
|  |  |  |
|  |  |  |
|  |  |  |
|  |  |  |
|  |  |  |
| GP verbal action post management plan | - Stops talking, listens to patient - Stops talking, works on computer - Continues talking, returns to treatment or investigation plan - Continues talking, discusses potential cause of symptoms - Continues talking, turns to computer - Concludes consultation - Starts to conclude consultation, then performs a patient examination - No video data |  |
|  |  |  |
|  |  |  |
|  |  |  |
|  |  |  |
|  |  |  |
|  |  |  |
| Patient posture post management plan | - Continues listening to GP - Dresses themselves - Sits while GP types on computer - No video data - Off camera - Produces a piece of paper/other object (e.g. inhaler), writes on paper - Returns to seat post examination - Speaks first post management plan - Stands up ready to leave |  |
|  |  |  |
|  |  |  |
|  |  |  |
|  |  |  |
|  |  |  |
|  |  |  |
| Patient verbal action post management plan | - Asks about logistics of accessing medication from pharmacy or secondary care or investigations - Asks about treatment - Discussion re how to contact patient - Reminds GP to weigh them - Returns to diagnostic meaning - Returns to discussing symptoms - Spouse speaks first - Thanks GP or starts to end consultation - Uses sarcasm - Silence - Makes small talk - Writes notes to self - Discusses management plan - No video data |  |
|  |  |  |
|  |  |  |
|  |  |  |
|  |  |  |
|  |  |  |
|  |  |  |
|  |  |  |
|  |  |  |
|  |  |  |
| Post management plan: patient final response | - Answers GP’s questions re medication - Discussion re treatment - Changes topic of discussion - Continues listening to GP - Continues symptom discussion - Continues writing notes - Discussion on summarising next steps - Dresses themselves - Moves on to next presenting problem - No video data - Patient asks about investigation results - Patient waits while GP works on computer - Starts to leave - Undergoes examination or assessment |  |
